# Ketones in Cardiovascular Health and Disease: An Updated Review

**DOI:** 10.3390/cells15020150

**Published:** 2026-01-14

**Authors:** Sanjiv Shrestha, Isis Harrison, Aminat Dosunmu, Ping Song

**Affiliations:** Institute for Biomedical Sciences, Georgia State University, 100 Piedmont Ave SE, Atlanta, GA 30303, USA; sshrestha7@student.gsu.edu (S.S.); iharrison5@student.gsu.edu (I.H.); adosunmu1@student.gsu.edu (A.D.)

**Keywords:** ketones, β-hydroxybutyrate, metabolic signals, cardiovascular disease, biomarkers, post-translational modification, ketone therapy

## Abstract

**Highlights:**

**What are the main findings?**

- Ketones function as alternative energy sources and signaling molecules that regulate cardiovascular health and diseases.
- The post-translational modification mediated by β-hydroxybutyrylation controls the fate and function of target proteins and their biological roles.

**What are the implications of the main findings?**

- Ketone intervention may serve as a promising therapeutic approach for cardiovascular diseases, such as heart failure, acute cardiac injury, and vascular dysfunction.
- Future ketone therapies should take into account the side effects of the ketogenic diet, as well as gender- and age-specific variations in the effectiveness of ketone treatments.

**Abstract:**

Ketones are metabolites primarily produced by the liver and are utilized by various organs outside of the liver. Recent advances have demonstrated that ketones serve not only as alternative energy sources but also as signaling molecules. Research indicates that ketones can influence cancer development and metastasis, cardiac metabolic and structural remodeling, physical performance, vascular function, inflammation, and the aging process. Emerging evidence from preclinical and early-phase clinical studies suggests that strategies such as ketone salts, ketone esters, and the ketogenic diet may offer therapeutic benefits for conditions like heart failure, acute cardiac injury, diabetic cardiomyopathy, vascular complications, atherosclerosis, hypertension, and aortic aneurysm. This literature review updates the current understanding of ketone metabolism and its contributions to cardiovascular health and diseases. We highlight the underlying molecular mechanism with post-translational modification known as β-hydroxybutyrylation, which affects the fate and function of target proteins. Additionally, we discuss the therapeutic challenges associated with ketone therapy, the potential of using ketone levels as biomarkers for cardiovascular diseases, as well as gender- and age-specific differences in ketone treatment. Finally, we explore future research directions and what is needed to translate these new insights into cardiovascular medicine.

## 1. Introduction

The major ketone bodies are D-β-hydroxybutyrate (β-OHB) and acetoacetate (AcAc), which can be converted into one another [1], along with trace amounts of acetone [2]. Among them, β-OHB is the primary circulating ketone body. During metabolic stress states, such as prolonged exercise, fasting, or carbohydrate deprivation, the synthesis of ketone bodies primarily occurs in the liver. In this process, acetyl coenzyme A, derived from fatty acid β-oxidation, serves as the main substrate [3]. The clinical significance of ketones became apparent with the discovery of diabetic ketoacidosis (DKA), a severe metabolic disorder in uncontrolled diabetes characterized by hyperketonemia, acidosis, and hyperglycemia [4]. In recent years, there has been a surge of scientific interest in ketones due to their diverse physiological roles and therapeutic implications, particularly in cardiovascular health [5]. β-OHB, specifically, has been identified as an efficient energy substrate, especially during endurance exercise or periods of low carbohydrate intake and caloric restriction. It enhances mitochondrial energy efficiency while preserving muscle glycogen [6]. In addition to its energy-providing role, ketones exhibit anti-inflammatory, antioxidant [7], and many other properties owing to their critical role as a signaling metabolite [2,8]. Notably, β-OHB functions as a signaling molecule by serving as a substrate for lysine β-hydroxybutyrylation (Kbhb) modification of target proteins [9,10]. This modification is involved in metabolite-regulated gene expression, DNA repair, protein stability, and metabolic remodeling [11]. These findings have led to increasing interest in exogenous ketone supplementation and ketogenic dietary interventions as potential strategies for enhancing physical performance [12], as well as for controlling cancer development and metastasis [13], neurodegenerative diseases [2,14], virus infections [15], immune response [16], and anti-aging [2]. As cardiovascular diseases (CVDs), including heart failure, acute myocardial infarction, atherosclerosis, peripheral artery disease, arrhythmias, and aortic aneurysms, remain leading causes of morbidity and mortality worldwide [17], there has been tremendous interest in the study of ketone metabolism within the cardiovascular context over the past several years [18,19]. Accumulating evidence suggests that ketone metabolism is upregulated in heart failure [20], providing a potential adaptive energy source for the failing myocardium [5,7,21,22]. In this review, we discuss recent research advances regarding ketones in cardiovascular health and disease, focusing on the most advanced areas of clinical interest and the protein post-translational modifications associated with β-hydroxybutyrylation. We also address potential adverse effects of ketones, particularly regarding ketogenic diets (KD), on cardiovascular systems, as well as gender differences in ketone therapy.

## 2. Overview of Ketone Metabolism in the Cardiovascular System

Ketone bodies are primarily produced through a process known as ketogenesis in the mitochondria of the liver and are subsequently released into circulation. Ketone bodies primarily enter cardiac muscle cells and vascular cells through plasma membrane monocarboxylate transporters (MCTs), specifically MCT1 and MCT2 [18]. MCT1 is upregulated in cardiomyocytes from congestive heart failure rats [23]. Interestingly, Koay et al. reported for the first time that the human heart has the inherent ability to generate ketones via the enzyme 3-hydroxy-3-methylglutaryl-coenzyme A synthase 2 (HMGCS2), which is a rate-limiting enzyme in ketogenesis [24,25]. Circulating ketones serve as an important source of metabolic fuel and signaling molecules for the heart and blood vessels, both under normal physiological conditions and during pathological states. Unlike glucose and fatty acid oxidation, the oxidation of ketones in cardiac tissues is not heavily regulated [26]. As a result, the rate of ketone oxidation in the cardiovascular system largely depends on their availability, which is closely tied to blood ketone levels [27]. Moreover, experimental evidence indicates that cardiac efficiency decreases when a healthy heart is exposed to elevated ketone levels (specifically at 2 mM β-OHB in ex vivo studies) [27]. Additionally, cardiac utilization of ketones is reduced in patients with type 1 diabetes (T1D) [28]. Furthermore, since circulating ketone levels are dynamic, they have recently been proposed as potential biomarkers for specific CVD [29,30].

### 2.1. Ketone Levels—Potential Biomarkers for Heart Disease

The levels of ketones in the body vary based on metabolic state. In healthy adults who are in a fed state, circulating ketone concentrations, as measured by a ketone meter, typically range from 50~250 µM [2]. When glucose levels drop due to starvation, ketone levels increase, entering a state known as light ketosis, which ranges from 0.6 to 1.5 mM. During prolonged fasting or extended periods of aerobic exercise, ketone levels can rise to about 3 mM, a state referred to as nutritional ketosis [31]. In contrast, in pathological conditions such as diabetic ketoacidosis, ketone levels may soar to as high as 20 mM. This extreme elevation is linked to metabolic acidosis and can lead to serious clinical emergencies [32] (Table 1).

Both plasma and myocardial concentrations of ketones are dramatically elevated in individuals with heart failure and reduced ejection fraction (HFrEF) [35,36]. Especially, the levels of 3-OHB and AcAc in plasma are increased in the severe HFrEF patients [37]. In contrast, circulating ketone levels do not seem to increase in patients with heart failure and preserved ejection fraction (HFpEF) [36,38]. Additionally, the levels of β-OHB and AcAc in the myocardium do not differ between control mice and those with HFpEF [39,40]. Conversely, fasting blood ketone levels are heightened across all mouse HFpEF mouse groups, with no significant additional increase in fasting ketone levels observed following treatment with empagliflozin [a sodium glucose co-transporter 2 inhibitor (SGLT2) inhibitor] or ketone ester [40].

Elevated blood ketone levels have been shown to correlate with the severity of cardiac dysfunction, highlighting their potential as prognostic indicators [41,42]. Studies indicate that circulating β-OHB is associated with an increased risk of HF and HFrEF in women, but not in men [43]. Additionally, elevated circulating AcAc is indicative of poor prognosis in HF patients [42]. Incorporating plasma ketones into a clinical risk score using biomarkers [N-terminal proB-type natriuretic peptide (NT-proBNP) and high-sensitivity troponin (hs-cTnT)] of cardiac injury and stress could enhance the prediction of incident HF [29]. Moreover, higher concentrations of ketones are linked to an increased risk of atrial fibrillation (AF) in mainly healthy, community-based cohorts [44]. In contrast, a reduction in β-OHB levels and citrate synthase activity may contribute to aging-related AF, suggesting these factors could serve as potential diagnostic biomarkers for this condition [45]. A recent retrospective study has shown that increased plasma β-OHB levels are an independent protective factor affecting the prognosis of cardiac function in patients with acute myocardial infarction (MI) combined with HF [46]. Additionally, another research demonstrates that elevated plasma β-OHB can predict adverse outcomes and disease progression in patients with arrhythmogenic cardiomyopathy [47]. Overall, these studies imply that plasma ketone levels may serve as valuable biomarkers for identifying populations at risk for cardiac diseases.

### 2.2. Ketone Supplementation and Ketogenic Diet

There are various approaches to stimulate ketogenesis and promote nutritional ketosis [48]. Ketone treatment can be executed either by following a ketogenic diet (KD), which is high in fat and low in carbohydrates, or by taking ketone supplements. These supplements may include ketone precursors such as 1,3-butanediol (BD) or medium-chain triglycerides (MCT), as well as exogenous ketones like ketone salts or ketone esters (KEs) [49,50] (Table 2). Among these options, KE seem to be the most effective and well-tolerated for achieving ketosis [50]. It is essential to determine target ketone levels and the best methods for safely and feasibly reaching and maintaining those levels over extended periods. Physiological ketosis can be achieved through various methods that alter fuel availability, such as calorie restriction or fasting. A KD increases the endogenous production of ketones to levels of 0.5–5 mM [51]. MCT oil provides medium-chain triglycerides that are rapidly converted into ketones, leading to a quick rise in ketone levels (0.5–2 mM) [52]. Exogenous ketone salts deliver β-OHB bound to minerals, resulting in a modest and temporary increase in ketone levels (0.5–1 mM) [53]. In contrast, ketone esters provide a stronger and quicker elevation of ketones (2–6 mM), although they tend to be more expensive and short-lived [48]. Finally, SGLT2 inhibitors can induce mild and sustained ketosis (0.3–1 mM) pharmacologically by reducing the reabsorption of glucose in the kidneys [18,54] (Table 2).

## 3. Updated Research Findings

Recent studies on ketones in the cardiovascular system have yielded significant findings. For instance, researchers have explored the unique oxidation of ketones in relation to cardiac disease and repair. Additionally, they have examined the anti-inflammatory properties of ketones, specific post-translational modifications such as β-hydroxybutyrylation, broader clinical trials, and the potential adverse effects of ketone therapy.

### 3.1. Ketones as an Energy Source in the Failing Heart

Heart failure is featured by a metabolic abnormality where there is a reduced capacity for the oxidation of primary fuels—fatty acids and glucose—leading to decreased cardiac efficiency [26]. Research suggests that in mice with HFpEF and diabetic cardiomyopathies, cardiac energy metabolism shifts from glucose oxidation to mitochondrial fatty acid oxidation [26,63]. In fact, fatty acid oxidation accounts for approximately 70% of adenosine triphosphate (ATP) formation in the hearts of HFpEF mice. Importantly, recent metabolomics studies have shown that failing heart significantly oxidizes β-OHB and AcAc, indicating a metabolic shift toward more energy-efficient substrates [34,64,65]. While it is well established that ketone oxidation is upregulated in heart with HFrEF [20,26], this does not lead to improvements in cardiac efficiency [66]. Conversely, a recent research found that the rise in ketone β-OHB oxidation rates is less pronounced in the hearts of HFpEF mice compared to control hearts. However, administering KE or using SGLT2 inhibitors can partially restore β-OHB oxidation rates in HFpEF hearts, though this restoration does not associate with improvements in cardiac function [40]. These findings suggest that myocardial ketone oxidation is impaired in aged female mice with HFpEF, which is induced by high-fat diet combined with the treatment of N^[w]^-nitro-l-arginine methyl ester L-NAME, an endothelial nitric oxide inhibitor [40]. This animal model is highly relevant to clinical conditions.

In HFrEF, there is an increase in the hepatic production of D-β-OHB, as well as elevated levels of L-β-OHB, which is specifically produced by epicardial adipose tissue (EAT). Ex vivo analyses of EAT explants indicate that in advanced HF, EAT undergoes significant metabolic remodeling characterized by impaired fatty acid oxidation and increased local production of L-β-OHB, which may help support cardiac energy needs in the failing heart [37]. Furthermore, in the Rotterdam study, β-OHB levels were found to be negatively correlated with EAT volume [67]. Recent research, including a randomized crossover study in pigs, has also examined the cardiovascular effects of these enantiomers. The study revealed that L-β-OHB resulted in greater improvements in hemodynamic parameters, such as cardiac output, compared to D-β-OHB [68].

### 3.2. Ketones in Acute Cardiac Injury—Myocardial Infarction and Ischemia–Reperfusion

Numerous studies have identified changes in fatty acid and glucose metabolism during acute cardiac injury; however, the profile and role of ketone metabolism in myocardial infarction (MI) and ischemia–reperfusion (I/R) remain largely unknown [69]. A post hoc analysis of the Empagliflozin in Acute Myocardial Infarction (EMMY) trial indicates that higher baseline β-OHB levels are inversely associated with cardiac function after an acute MI [70]. Furthermore, circulating ketone levels increase in patients presenting with ST-segment elevation myocardial infarction (STEMI). Elevated ketone levels 24 h after reperfusion are linked to poorer functional outcomes following STEMI, suggesting a potential role for ketones in response to myocardial ischemia [71]. In acute MI, elevated blood AcAc levels reduce the risk of major adverse cardiac and cerebrovascular events (MACCE) [72]. In individuals recovering from MI, ketones provide a supplemental energy source for both the heart and the vasculature [19]. In a swine model of acute MI, oral KE supplementation stimulates myocardial β-OHB extraction in both healthy and infarcted hearts. Additionally, oral KE supplementation improves cardiac substrate uptake and utilization, boosts cardiac ATP generation, and decreases cardiac inflammation after MI [73] (Table 3). In a nondiabetic porcine model of I/R, intravenous infusion of β-OHB during myocardial ischemia results in significantly higher myocardial salvage, smaller MI size, less microvascular obstruction, and improved cardiac function, as evidenced by metrics such as left ventricle ejection fraction and strain [74]. In male mice, a single dose of β-OHB treatment via intraperitoneal injection at the onset of reperfusion decreases infarct size, maintains cardiac function, and ultimately alleviates myocardial I/R injury [75]. Interestingly, the SGLT2 inhibitor empagliflozin also demonstrates cardioprotective effects that may be mediated through β-OHB induction during acute MI [74]. Additionally, β-OHB treatment has been shown to improve heart function and stimulate angiogenesis after MI in mice by promoting lysine β-hydroxybutyrylation of HIF prolyl hydroxylase 2 (PHD2) at lysines 239 and 385 [76]. Recent research by Hørsdal et al. demonstrated that intravenous infusion of β-OHB increases cardiac contractility and reduces vascular resistance in a porcine model of cardiogenic shock caused by acute MI [77] (Table 3). Collectively, these studies suggest that ketones may enhance recovery from MI or ischemia–reperfusion injury.

### 3.3. Ketones and Vascular Complication

The vasculature is emerging as a critical target for ketones. In this update, we review preclinical research on the effect of ketones in vascular diseases. It has been reported that β-OHB binds to its receptor, G-protein-coupled receptor 109a (Gpr109a). This interaction promotes the influx of extracellular calcium (Ca^2+^), which reduces the release of Ca^2+^ from the endoplasmic reticulum (ER) to the mitochondria, thus inhibiting ER stress in macrophages [81]. As a result, β-OHB blocks the activation of the NOD-like receptor family, pyrin domain containing protein 3 (NLRP3) inflammasome, leading to a reduction in the proportion of pro-inflammatory M1 macrophages and an increase in cholesterol efflux, which dramatically alleviates atherogenesis in mice [81]. Unlike β-OHB, AcAc has been shown to have harmful effects on blood vessels. Experimental studies conducted on human umbilical cord vascular endothelial cells (HUVECs) demonstrated that elevated levels of AcAc increase oxidative stress and adhesion between endothelial cells and monocytes [92]. Additionally, AcAc was found to promote inflammatory signaling by increasing IL-6 in human monocytes. However, another study indicated that AcAc might provide protective effects against mitochondrial dysfunction induced by lactic acidosis in macrophages [93].

Additionally, Lan et al. recently showed that treatment with 1,3-butanediol, a precursor of β-OHB, mitigates aortic calcification in rats with chronic kidney disease and in mice overloaded with VitD3 by reducing HDAC9 in vascular smooth muscle cells (VSMCs) [84]. Supplementation of 1,3-butanediol in drinking water also attenuates hypertension and protects kidney function in high-salt diet rats [94]. Furthermore, β-OHB has been shown to prevent vascular cell senescence in mouse aorta [95]. Interestingly, ketosis induced by a KD or exogenous ketones reduces the levels of C–C chemokine receptor type 2 (CCR2) in the aorta, maintains the balance of matrix-metalloproteinase (MMP), inhibits extracellular matrix (ECM) degradation, and resultantly prevents abdominal aortic aneurysm rupture in male Sprague–Dawley rats [91]. Overall, these findings suggest that ketones may offer both preventive and therapeutic effects on vascular diseases (Table 3).

### 3.4. Anti-Inflammatory and Antioxidant Effects

Inflammation and oxidative stress play a central role in the development of CVD. Ketones have been reported to regulate both inflammation and oxidative stress [14,96]. For instance, increasing levels of β-OHB through KE treatment reduces the formation of NLRP3 inflammasome and counteracts mitochondrial dysfunction triggered by pro-inflammatory cytokine. Furthermore, β-OHB downregulates the acetyl-CoA pool, partially by activating citrate synthase and inhibiting fatty acid uptake, which in turn inhibits the mitochondria-inflammation cascade [97]. As a result, KE, through the production of β-OHB, improves heart function in HFpEF mouse model established using the 3-Hit strategy [97]. Liao et al. also found that a weekly intraperitoneal injection of β-OHB slows the progression of HFpEF in mice by increasing cardiac CD3^+^CD4^+^Foxp3^+^ Treg cells through modulation of the antioxidant pathway involving NADPH oxidase 2 (NOX2) and glycogen synthase kinase-3β (GSK3β) [98]. In addition, KE supplementation has been shown to reduce inflammatory markers, oxidative stress, and apoptosis in swine cardiac tissues following MI [73]. These studies collectively indicate that β-OHB has anti-inflammatory and antioxidant properties. However, the effects of KD on inflammation remain controversial. More research is needed to investigate the anti-inflammatory effects of β-OHB on endothelial injury and vascular remodeling in conditions such as atherosclerosis and hypertension.

### 3.5. Updated Molecular Mechanism with β-Hydroxybutyrylation

Ketones, especially β-OHB, have a significant impact on the heart and blood vessels, functioning as signaling molecules that regulate post-translational modifications as well as gene transcription and translation [99]. This regulation leads to various cellular responses [25]. One important modification is lysine β-hydroxybutyrylation (Kbhb), which influences the fate and activity of many proteins in both physiological and pathological contexts, particularly within the cardiovascular system and cancer (Table 4). Recent studies in cardiovascular cells suggest that β-hydroxybutyrylation may govern the expression of genes related to angiogenesis [100], smooth muscle contractility, antioxidant defense, and fatty acid metabolism [101]. The specific context in which this modification occurs emphasizes its potential dual role in cardiovascular repair and pathology during prolonged periods of ketosis. Therefore, further investigation into its genomic targets in cardiovascular tissues is necessary. In addition, β-OHB is an antagonist for free fatty acid receptor 3 [FFAR3, also known as GPR41 (G-protein-coupled receptor 41)] [102]. Thus, it may play critical roles in cardiac pathophysiology via neuronal norepinephrine release [103].

### 3.6. Clinical Trials of Ketone Treatment

Accumulating evidence from both animal and human research suggests that ketones may provide preventive or therapeutic benefits for patients with CVD [5,48,112]. Several smaller clinical trials involving short-term ketone treatment have been conducted [3,21]. For example, in the KETO-CHF trial (NCT05161650), a 2-week oral KE treatment for HFrEF patients demonstrated that chronic KE treatment enhances cardiac output (CO) and lowers cardiac filling pressures both at rest and during exercise [85]. In a substudy of the KETO-CHF trial, the 14-day KE treatment for patients with HFrEF results in elevated levels of renin and aldosterone, decreased levels of NT-proBNP and erythropoietin (EPO), and reduced circulating iron availability. These findings imply that sustained KE treatment influences neurohormonal regulation and fluid balance in HFrEF patients [113]. In another clinical trial (NCT05236335) involving patients with HFpEF and type 2 diabetes (T2D), a 2-week oral KE treatment (25 g of D-β-hydroxybutyrate-(*R*)-1,3-butanediol taken four times daily), followed by a 2-week washout period, stimulates CO and reduces cardiac filling pressures and ventricular stiffness. At peak exercise, KE treatment dramatically decreases pulmonary capillary wedge pressure and improves pressure-flow relationship [86]. Additionally, a clinical trial (NCT05768100) with oral 1,3-butanediol for HFrEF patients results in prolonged ketosis and subsequently increased CO and left ventricular ejection fraction [114]. Another study (NCT03560323) administering infusions of three different doses of β-OHB for patients with T2D and HF showed a dose-dependent increase in CO, left ventricular ejection fraction, and myocardial blood flow, without affecting myocardial glucose uptake [115]. A clinical trial (NCT04615754) investigating the use of ketones for pulmonary hypertension has shown that β-OHB infusion improves CO and reduces pulmonary vascular resistance in patients with pulmonary hypertension or chronic thromboembolic pulmonary hypertension [80]. Most recently, another clinical trial (NCT04656236) assessed the effects of a 3 h infusion of β-OHB or tonicity-matched saline, with a 1 h washout period, on patients with T1D and healthy controls. This study demonstrated that individuals with T1D have an impaired cardiovascular response to β-OHB, resulting in less induction of CO [28]. Furthermore, an ongoing clinical trial (NCT06715748) is examining the effects of exogenous ketones on acute changes and recovery of heart muscle after intense exercise. Collectively, these findings suggest that regulating circulating ketone levels may offer a novel treatment approach for patients with cardiopulmonary diseases. While exogenous ketones have been shown to lower blood pressure [94], and ameliorates conditions such as atherosclerosis [81], vascular calcification [84], and abdominal aortic aneurysm [91] in the preclinical studies, there remains a significant gap in translating these findings to patient outcomes [3], as no studies have directly assessed the effects of ketones on patients with atherosclerosis, hypertension, or aneurysmal disease.

## 4. Challenges of Ketone Therapies and Safety Considerations

Although the preclinical and clinical research highlights the impressive therapeutic benefits of ketones for CVD [21], multiple studies indicate potential harmful effects on the cardiovascular system. The impact of the KD on overall mortality and CVD mortality remains controversial. For instance, recent research suggests that KD may reduce all-cause mortality without increasing cardiovascular-related mortality in the adult population of the United States [116]. Recent studies have also found that ketones can affect cardiac fibrosis [117]. On one hand, treatment with β-OHB has been shown to restore mitochondrial function and mitigate heart interstitial fibrosis in mice with post-ischemic heart injury [118]. Additionally, the compound KE ((*R*)-3-hydroxybutyl-(*R*)-3-hydroxybutyrate) significantly decreases cardiac fibrosis in mice that underwent transverse aortic constriction surgery [119]. The KD has been reported to diminish right ventricular fibrosis in male Sprague–Dawley rats suffering from right ventricular failure induced by monocrotaline (MCT) [120]. On the other hand, prolonged KD treatment (8 to 16 weeks) can inhibit mitochondrial function and lead to heart fibrosis in healthy rats [87] and mice [89]. KD treatment lasting 4 to 8 weeks also increases cardiac fibrosis in adult spontaneously hypertensive rats [121] and HF mice [122]. Moreover, the KD has been associated with pro-inflammatory effects, such as exacerbating colitis in mice [123]. It can also trigger p53-mediated cell senescence in mouse hearts, whereas the intermittent KD helps prevent cell senescence [88]. A recent report indicated that a long-term KD (almost one year) leads to hyperlipidemia, liver dysfunction, severe glucose intolerance, and impaired insulin secretion in mice [124]. Even short-term treatment (6 weeks) with KD can result in metabolic syndrome characterized by body weight gain, hyperlipidemia, and lipid accumulation in the heart and liver [125]. However, the long-term benefits and risks of KD concerning the cardiovascular system remain largely unexplored. Furthermore, the underlying mechanisms for the varying outcomes in different studies are still unclear.

Of note, recent studies highlight that ketone treatment yields sex- and age-specific responses. For instance, both younger and older male mice on a KD tend to develop glucose intolerance. In contrast, the KD acutely improves rotarod performance in younger female mice [125]. Additionally, histone modifications and circulating β-OHB levels in Viable Mottled/Dunn and Kendall (VM/Dk) mice show distinct metabolic responses to the KD (Teklad KD2) [126]. A recent research indicates that male mice on the KD, which is characterized by high fat, low carbohydrates, and low protein, experience weight loss accompanied by reductions in both fat and lean mass, along with increased insulin sensitivity. Interestingly, these male mice display similar circulating levels of β-OHB as female mice. Conversely, female mice on the KD tend to gain fat mass and body weight, ultimately developing glucose intolerance and insulin resistance [127]. Moreover, when examining specific aspects of cardiac metabolism, a crucial distinction arises between supplement strategies (such as ketones) and dietary strategies that affect ketogenesis [128]. It is also important to recognize that individual responses to ketosis can vary, making long-term adherence to the KD challenging. Additionally, many of these therapies still require further basic and clinical investigations to ensure their safety and efficacy. Long-term feasibility of β-OHB use, along with the risk of ketoacidosis, presents further challenges for the sustained application of ketone treatments. Therefore, extensive long-term clinical trials are necessary to fully evaluate the safety, efficacy, and cardiovascular implications of ketones.

## 5. Conclusions and Future Directions

Ketones play important roles in energy metabolism and signaling within the cardiovascular system through various mechanisms, including the β-hydroxybutyrylation of specific proteins involving lysine. Growing evidence suggests potential benefits of ketones for conditions such as heart disease, cardiac injury, and vascular diseases. However, ketone treatments may also have detrimental effects on the cardiovascular system. To translate these novel findings into clinical applications, several investigations are necessary: (1) Conduct long-term, large-scale clinical trials to assess the cardiovascular efficacy, safety, and durability of oral ketone supplementation or KD in patients; (2) Investigate the sex- and age-specific differences observed in mice [125,126,127,129,130] when using KD, and carry out contextual and individual optimization research for ketone therapy; (3) Develop treatment methods that maintain sustainable ketone levels with less frequent dosing and formulations that are more palatable, which are essential for progressing to larger Phase 2 and 3 trials [35]; (4) Explore how ketones influence different types of cell death in cases of myocardial ischemia–reperfusion injury [131]; (5) Analyze the cell-specific mechanisms of β-OHB signaling, particularly in endothelial cells, smooth muscle cells, and resident vascular immune populations, to identify potential therapeutic windows; (6) Integrating omics approaches, such as metabolomics and epigenomics, along with advanced imaging and cardiovascular functional testing, could provide personalized insights into which individuals might benefit the most from ketone modulation; (7) Monitor body weight and metabolic parameters, as the potential impact of ketones on cardiovascular system may be through their effects on metabolism and endocrine system [132,133,134].

## Figures and Tables

**Table 1:** Blood ketone levels and their interpretations.

Ketone Level (mM)	Physiological/Pathological States	Interpretation	Recommended Action	Reference
≤0.5	Healthy adults with normal feeding	Baseline ketone production	None required	[25]
0.6–1.5	Early fasting/post-prolonged exercise/light ketosis	Slightly elevated; monitor if diabetic	Retest in 2 h; for diabetic individuals, consult a healthcare provider if symptoms present	[14,32]
1.6–3.0	Prolonged fasting/post-ultra endurance efforts/optimal nutritional ketosis	Moderate elevation; potential DKA risk in diabetics	For diabetic individuals, seek medical advice; monitor closely	[5,6,14]
3.0–6.0 *	Nutrition-provoked ketosis/early stage of pathological ketosis	Marked elevation; high risk of DKA * when accompanied by hyperglycemia	Immediate medical attention required if symptoms of DKA are present	[2,5,33,34]
>6.0	Severe DKA	Pathological and life-threatening ketoacidosis	Immediate medical emergency	[25,32]

* While blood ketone levels > 3 mM may indicate potential risks of DKA, ketone levels in DKA patients may reach as high as 20 mM.

**Table 2:** Features of ketone supplements and ketogenic diet.

Approaches	Depth of Ketosis (β-OHB Levels)	Key Characteristics	Common Applications	Reference
Exogenous ketone salts	Mild: 0.5–1 mM	Rapid but transient effect; less potent than esters; may cause GI discomfort or mineral load.	Performance boost and experimental therapeutic use.	[55,56,57,58]
Exogenous ketone esters	Moderate to high: 2–6 mM	Strong, rapid rise in ketones; more potent than salts; expensive and less palatable; short-lived effect without carb restriction.	Cognitive enhancement, therapeutic research, performance boost.	[55]
Ketone precursor 1,3-butanediol	Mild: 0.3–0.8 mM	Gradual, transient rise in ketones; GI discomfort, nausea, dizziness, ethanol-like side effects.	Therapeutic research.	[59]
Ketone precursor MCT Oil	Mild to moderate: 0.5–2 mM	Rapid ketone rise; supportive of ketogenic diet; high doses may cause GI distress.	Enhancing ketosis, athletic and cognitive support.	[52,60]
Ketogenic diet	Mild to moderate: 0.5–5 mM	Sustainable long-term with adherence; fat composition influences health outcomes; GI discomfort.	Weight management, metabolic syndrome, epilepsy.	[51,61]
SGLT2 Inhibitors (medication)	Mild: 0.3–1 mM	Pharmacological induction of low-level ketosis; sustained effect but shows multiple physiological effects; rare risk of ketoacidosis.	Management of type 2 diabetes and heart failure.	[18,54,62]

**Table 3:** Key effects of ketones on cardiovascular system.

Ketones or Related Treatments	Targeted Organs or Cells	Outcomes	Reference
Caloric restriction or β-OHB	Heart and macrophages of mouse	Promotes neovascularization and cardiac repair following myocardial infarction in mice	[76]
β-OHB	Human hearts with HFrEF	β-OHB infusion increases cardiac output	[66]
Na-β-OHB	Rat heart and blood vessel	Increases cardiac contractility and lowers systemic vascular resistance resulting in elevated cardiac output	[78]
β-OHB	Heart of female pigs with cardiogenic shock	Intravenous β-OHB infusion increases cardiac contractility and reduces vascular resistance resulting in elevated cardiac output	[77]
β-OHB	Rat heart; cardiac fibroblasts and macrophages	Reduces cardiac fibrosis in diabetic cardiomyopathy; Encourages M1 to M2 macrophage reprogramming	[79]
β-OHB	Pulmonary hypertension patients and Sprague–Dawley rats (heart and pulmonary arteries)	β-OHB infusion increases cardiac output and reduces pulmonary vascular resistance	[80]
β-OHB	Macrophage and mouse aorta	Daily oral treatment with β-OHB decreases the M1 macrophage proportion and attenuates atherosclerosis in mice	[81]
β-OHB	Brain and aorta of mice	Reduces atherosclerotic plaque formation; reduces lipid deposition in the choroid plexus in the brain	[82]
β-OHB	Mouse aorta and rat VSMC	Daily gavage of β-OHB alleviates atherosclerotic calcification and reduces endoplasmic reticulum stress and stress-mediated apoptosis in mice aorta	[83]
1,3-butanediol	Arterial tissue and VSMC	Daily gavage of 1,3-butanediol decreases HDAC9 in VSMC and restrains aortic calcification in CKD rats and VitD3-overloaded mice	[84]
Ketone ester	Heart of patient with HFrEF	Elevates cardiac output and decreases cardiac filling pressures at rest and during exercise	[85]
Ketone ester	Heart of patient with both HFpEF and T2DM	Reduces cardiac filling pressures in patients with HFpEF	[86]
Ketone ester	Heart of a swine model of acute myocardial infarction	Oral ketone ester enhances the myocardial consumption of β-OHB and fatty acid and inhibits cardiac inflammation	[73]
Prolonged ketogenic diet or β-OHB	Rat cardiomyocytes, rat heart, or human atrial fibrillation heart tissue	Decreases mitochondrial biogenesis and increases cardiomyocyte apoptosis and cardiac fibrosis	[87]
Ketogenic diet	Mouse heart and kidney	Induces p53-dependent cellular senescence in mouse heart and kidney	[88]
Ketogenic diet (with long and medium-chain fatty acids)	Mouse heart tissue	Induces cardiac fibrosis in adult male mice	[89]
Ketogenic diet	Mouse aorta and macrophages	Reduces plaque size in aldosterone-induced atherosclerosis in ApoE −/− mice; encourages M1 to M2 inflammatory profile switch	[90]
Ketogenic diet	Abdominal aorta of male Sprague–Dawley rats	Decreases CCR2 levels, inhibits ECM degradation, reduces AAA expansion and incidence of rupture	[91]

**Table 4:** Major proteins modified by β-hydroxybutyrate.

Target Proteins	Modified Amino Acid	Experimental Models	Biological Outcomes	Reference
HIF prolyl hydroxylase 2 (PHD2)	Lysine 239 and 385	Macrophage and MI induced by ligation of the left anterior descending coronary artery	Inhibits PHD2 activity and recovers postinfarction cardiac function by enhancing neovascularization	[76]
Citrate synthase (CS)	Lysine 395	H9C2, HEK293T cells, and heart of KE-treated HFpEF mouse	Increases CS activity and downregulates the acetyl-CoA pool, mitochondrial acetylation, and subsequent inflammation	[97]
Histone 3	Lysine 9	Endothelial cells and myocardial infarction	H3K9bhb-enhanced chromatin opening promotes transcription of the proangiogenic genes, accelerating hypoxic endothelial angiogenesis post-MI	[100]
Histone 3	Lysine 9 and 18	HEK293, HCT116, MEF cells, and mouse liver	Upregulates genes in starvation-responsive metabolic pathways. H3K9bhb is enriched in active gene promoters and is associated with genes upregulated in the starvation-responsive pathway	[9,10]
Histone 4	Lysine 8	HCT116, HEK293, and MEF	Mediates transcription in vitro	[10]
P53	Lysine 120, 319, and 370	U2OS, HCT116 cells, and thymus tissues of fasted mice	Attenuates p53 activity and decreases cell growth arrest and apoptosis	[104]
Atp5f1a	Lysine 239	Myocardial tissues in a mouse model of Diabetic cardiomyopathy (DbCM)	Restore mitochondrial function in alleviating diabetic cardiomyopathy	[105]
Succinate-CoA ligase subunit alpha (SUCLG1)	Lysine 393 in CS and lysine 81 in SUCLG1	APP/PS1 Alzheimer’s mouse model	Promotes enzymatic activities of CS and SUCLG1 and ATP production, but also attenuates β-amyloid plaque pathologies and microgliosis in APP/PS1 mice	[106]
Potassium channel tetramerization domain containing 9 (KCTD9)	Lysine 123 and 129	Human colorectal cancer cell lines and nude mice	Mediates the ubiquitination and degradation of KCTD9 and enhances the progression and metastasis of colorectal carcinoma	[107]
Snail	Lysine 152	Human PDAC cell lines PANC-1 and SW1990 and BALB/c nude mice	Increases Snail stability and promotes pancreatic cancer cell metastasis	[13]
Calcium/calmodulin-dependent kinase II-α (CaMKII-α)	Lysine 42 and 267	Male mice with type 1 diabetes mellitus (T1DM)	Inhibits hippocampal CaMKII activity and induces memory deficits in mice with T1DM	[108]
Superoxide dismutase 2 (SOD2)	Lysine 68	Mouse macrophage	β-OHB stabilizes SOD2 protein and ameliorates lipopolysaccharide-induced liver injury in mice	[109]
3-oxoacid CoA-transferase 1 (OXCT1)	Lysine 421	Cultured cells and fasted and T1D mice	Increases OXCT1 enzymatic activity and accelerates ketone body utilization	[110]
Signal transducer and activator of transcription 1 (STAT1)	Lysine 679 (major), 193, 286, 336, 379, 652	Mouse bone marrow-derived macrophages and other cell lines	Inhibits M1 macrophage polarization by reducing STAT1 phosphorylation and transcriptional activity	[111]

## Data Availability

No new data were created or analyzed in this study.
